# A Shared Systemic Metabolic Signature Across the Neurological Disease Spectrum: A Summary-Level Analysis of 274,241 UK Biobank Participants

**DOI:** 10.3390/biomedicines14081773

**Published:** 2026-08-06

**Authors:** Likun Yang, Wei Lin, Seyed M. Mirsattari

**Affiliations:** 1 Department of Neurosurgery, The 904th Hospital of the Joint Logistics Support Force of the People’s Liberation Army, Wuxi 214044, China; 2 Department of Neurologic Surgery, Mayo Clinic Florida, Jacksonville, FL 32224, USA; 3 Department of Neurology, Mayo Clinic Florida, Jacksonville, FL 32224, USA

**Keywords:** pre-diagnostic, metabolomics, Mendelian randomization, UK Biobank, systemic metabolism, neurological metabolite score

## Abstract

**Background:** Neurological diseases share overlapping systemic and molecular features, but the specificity and interpretation of cross-disease metabolomic signatures remain uncertain. **Methods:** We analyzed summary-level Nightingale NMR metabolomic and genetic data from 274,241 UK Biobank participants across eight neurological endpoints. The disease rankings used here were derived from baseline plasma metabolites predicting future incident disease rather than from contemporaneous diagnostic case–control contrasts. **Results:** Among 57 priority metabolites with genome-wide significant instruments (2472 SNPs; mean F = 162.4), nine ranked in the top 30 for at least seven of the eight endpoints, defining a shared pre-diagnostic neurological signature confirmed as non-random cross-endpoint convergence by permutation testing (*p* < 0.0001). Because we did not perform a quantitative non-neurological disease control analysis, this signature should not be interpreted as neurologically specific and may partly reflect systemic morbidity, renal function, body composition, or frailty-related physiology. Forward Mendelian randomization across 45 metabolite–disease pairs found no Bonferroni-significant causal effects; three nominal protective associations are consistent with the number of false-positive findings expected under multiple testing and require replication. **Conclusions:** These results support a shared systemic, pre-diagnostic metabolic signature across the neurological disease spectrum, while the null MR findings and specificity limitations favor interpretation as a biomarker or prodromal downstream signal rather than a proven causal mechanism. External prospective validation is essential.

## 1. Introduction

Neurological disorders relevant to this study include neurodegenerative diseases such as Alzheimer’s disease (AD), Parkinson’s disease (PD) [1], and amyotrophic lateral sclerosis (ALS) [2], together with inflammatory demyelinating and vascular cognitive disorders such as multiple sclerosis (MS) and vascular dementia. Despite distinct clinical phenotypes and proteinopathies, converging evidence from neuropathology, genetics, and imaging suggests that these conditions share overlapping pathobiological mechanisms. Co-pathology studies have demonstrated that amyloid, tau, alpha-synuclein, and TDP-43 aggregates frequently co-occur within the same brain, often in proportions that defy single-disease classification [3]. Recent multi-disease subtyping analyses using large-scale biobank data have further reinforced the view that neurodegeneration exists on a biological continuum rather than as discrete entities [4]. These observations raise a fundamental question: if the downstream proteinopathies converge, do the upstream metabolic perturbations converge as well?

The emergence of high-throughput metabolomic profiling in population-based biobanks offers an unprecedented opportunity to address this question. The UK Biobank, with Nightingale Health nuclear magnetic resonance (NMR) spectroscopy data on over 274,000 participants and with a median follow-up of 14.9 years, provides the scale and breadth required for systematic cross-disease metabolomic comparison [5]. Nightingale NMR quantifies 313 metabolites and derived ratios spanning amino acids, lipids, fatty acids, ketone bodies, and glycolysis intermediates—many of which have established links to neurodegeneration in smaller, single-disease studies [6,7]. You et al. [5] mapped the plasma metabolome to 527 prevalent and 859 incident diseases, revealing that metabolite profiles predict disease risk with accuracy comparable to established clinical biomarkers. Qiang et al. [8] characterized the genetic architecture of the plasma metabolome across 254,825 individuals, providing the genome-wide significant metabolite instruments that enable downstream Mendelian randomization. Together, these datasets provide the raw material for a systematic cross-disease metabolomic analysis that was previously infeasible.

Despite this wealth of data, no study has systematically asked which metabolites are shared across the neurological disease spectrum examined here. Existing Mendelian randomization studies of metabolites and neurodegeneration have focused on individual disease–metabolite pairs, typically testing a handful of candidate metabolites against a single outcome [9,10,11,12]. This disease-by-disease approach misses the possibility that a common metabolomic fingerprint underlies multiple neurological endpoints. The absence of a cross-disease framework also means that shared pathways—such as the well-established cerebral glucose hypometabolism observed as the earliest detectable abnormality across AD, PD, and ALS [6]—remain siloed within disease-specific studies. A cross-disease metabolomic signature, if validated, could, in principle, inform disease-agnostic risk stratification in pre-symptomatic populations; however, this remains a long-term motivation rather than a demonstrated clinical application. The present study did not evaluate risk-stratification performance (no AUC, calibration, or held-out validation); such clinical utility remains to be established. Furthermore, by applying Mendelian randomization to such a signature, one can evaluate whether the shared metabolomic associations reflect causal upstream perturbations or are merely consequences of neurodegeneration-related physiological changes.

**Figure 1:**
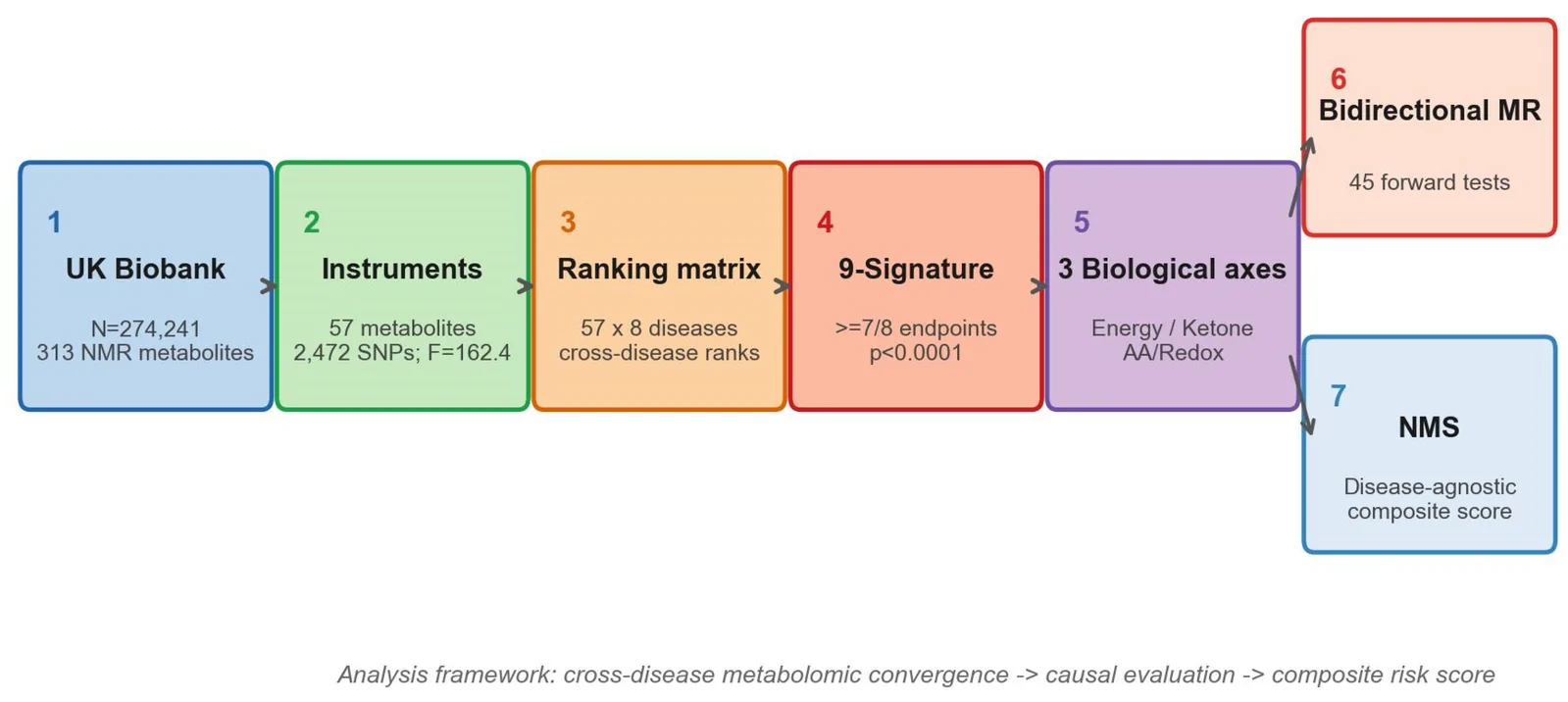
Study overview and analytical workflow. Schematic summarizing the three-aim analytical framework applied to 274,241 UK Biobank participants with Nightingale Health NMR metabolomic profiling. Aim 1 (Priority Metabolite Selection): identification of 57 priority metabolites with genome-wide significant genetic instruments (2472 SNPs; mean F-statistic = 162.4) from the NMR metabolomic GWAS, stratified into Tier 1 (*n* = 18), Tier 2 (*n* = 19), and Tier 3 (*n* = 20). Aim 2 (Cross-Disease Signature Discovery): construction of the 57 × 8 metabolite–disease-ranking matrix across 8 neurological endpoints and identification of the nine-metabolite shared systemic signature via a pre-specified coverage threshold (≥7/8 disease endpoints, top-30 rank), assessed by permutation testing (*p* < 0.0001). Aim 3 (Causal Inference and NMS Derivation): forward Mendelian randomization across 45 metabolite–disease pairs and derivation of the disease-agnostic Neurological Metabolite Score (NMS) using inverse-rank weighting. Arrows indicate the direction of analytical flow from data inputs to key outputs.

Here, we report a systematic cross-disease metabolomic analysis of 57 priority metabolites across eight neurological endpoints in 274,241 UK Biobank participants (Figure 1). Using 2472 genome-wide significant genetic instruments (mean F-statistic = 162.4), we identify a nine-metabolite signature that appears in the top 30 predictive metabolites for at least seven of the eight disease endpoints—a pre-specified threshold designed to capture repeated pre-diagnostic occurrence across the neurological endpoints analyzed. We demonstrate that this cross-disease convergence significantly exceeds chance expectation via permutation testing, organize the metabolites into three interpretive biochemical groupings (energy-related, ketone-related, and amino acid/redox-related markers), and perform forward Mendelian randomization with Steiger directionality filtering to evaluate causal relationships. Finally, we derive a summary-level Neurological Metabolite Score (NMS) that synthesizes the signature into a single composite metric applicable to cohorts with Nightingale NMR data, while emphasizing that it remains unvalidated and hypothesis-generating. Our findings support a shared systemic metabolic signature associated with future neurological disease risk, with specificity and causal boundaries explicitly limited by the absence of non-neurological controls and by null forward MR results.

## 2. Materials and Methods

### 2.1. Data Sources, NMR Platform, and Priority Metabolites

This summary-level study used UK Biobank metabolomic and genetic resources from previously published analyses [5,8]. The UK Biobank is a prospective population-based cohort of approximately 500,000 adults aged 40–69 years at recruitment (2006–2010) [5]. Plasma metabolomic profiling was available for 274,241 participants with Nightingale Health NMR (Helsinki, Finland) spectroscopy data. The median follow-up duration in the source phenome analysis was 14.9 years, during which incident neurological disease diagnoses were ascertained through linked hospital inpatient records, death registries, and primary care data.

We examined eight neurological disease endpoints for the ranking analysis: seven non-epilepsy neurological endpoints—Parkinson’s disease (PD; FinnGen G6_PARKINSON), PD strict definition (PDSTRICT_EXMORE), Alzheimer’s disease (AD; FinnGen G6_ALZHEIMER), Alzheimer-type dementia (F5_ALZHDEMENT), vascular dementia (F5_VASCDEM), amyotrophic lateral sclerosis/motor neuron disease (ALS; FinnGen G6_ALS), and multiple sclerosis (MS; FinnGen G6_MS)—together with epilepsy (G6_EPLEPSY) as an additional neurological comparator (Section 2.3). The FinnGen endpoint codes above define the disease columns of the ranking matrix; the independent large-consortium GWAS used as outcome datasets for forward Mendelian randomization are specified separately in Section 2.6. Frontotemporal dementia (FTD) and Lewy body dementia (LBD) were excluded due to insufficient case counts (N < 150 each) in the UK Biobank, which would preclude reliable metabolomic profiling.

For the forward Mendelian randomization analyses (Section 2.5), disease-outcome genome-wide association study (GWAS) summary statistics for the five primary neurological disease outcomes were obtained from published large-scale consortia: PD from Nalls et al. [9] (MR-Base ID: ieu-b-7), AD from Bellenguez et al. [10] (ebi-a-GCST90027158), ALS from Nicolas et al. [11] (ebi-a-GCST005647), MS from the International Multiple Sclerosis Genetics Consortium [12] (ieu-b-18), and vascular dementia from FinnGen (finn-b-F5_VASCDEM).

Metabolomic data and metabolite–disease rankings were derived from You et al. [5], and genetic instrument data were derived from Qiang et al. [8], with the genetic summary data deposited in the Figshare repository (DOI: 10.6084/m9.figshare.29390471). The two source studies are comparable in that both use the UK Biobank Nightingale NMR platform and the same 313-feature metabolomic measurement framework; however, they address different inferential targets. You et al. provide baseline metabolite-to-phenome associations and incident-disease prediction rankings, whereas Qiang et al. provide genetic architecture and GWAS instruments for metabolic traits. We therefore use You et al. for disease-ranking evidence and Qiang et al. for MR instrument construction, without treating their models, covariate structures, or endpoint definitions as interchangeable.

Data access clarification: This study exclusively utilized summary-level data. No individual-level UK Biobank data were accessed for the primary analyses reported here; accordingly, no UK Biobank Application ID is required for the analyses presented. We inspected a local UK Biobank individual-level covariate export available to the authors, which contained demographic, anthropometric, lifestyle, blood biochemistry, genetic QC/principal-component, medication/self-reported illness, and hospital-/death-record fields. However, this local export did not contain the complete Nightingale NMR metabolomics panel or the disease-by-metabolite ranking matrix required to reproduce the You et al. rankings, perform quantitative non-neurological control analyses, or re-estimate NMS disease-specific weights. We therefore did not use this individual-level export for partial covariate adjustment: the primary analytic unit in this manuscript is the summary-level metabolite-by-disease-ranking matrix generated in the original full-panel, source-model framework, and mixing that framework with a different, incomplete individual-level export would produce estimates that are not comparable with the rankings used for signature discovery. Source-model covariate adjustment therefore follows the original reports; residual differences in adjustment for factors such as BMI, renal function, fasting or sampling state, medication, and lifestyle variables remain inherited limitations of this summary-level analysis.

Plasma metabolites were quantified using the Nightingale Health high-throughput NMR spectroscopy platform, which measures 313 metabolites and derived ratios from a single 350 uL plasma sample [5,8]. The platform covers lipoprotein subclasses, fatty acids, amino acids, ketone bodies, fluid balance markers, and glycolysis/gluconeogenesis intermediates. Use of the same NMR platform improves metabolite panel comparability across the source studies, but it does not remove differences in disease-model construction, covariate adjustment, or downstream analytic purpose.

For downstream Mendelian randomization analyses, metabolite-level GWAS summary statistics were generated from the UK Biobank NMR data (*N* = 274,241), as previously described [5,8]. The metabolomic data were rank-inverse normal transformed prior to GWAS to ensure normality of the test statistics.

### 2.2. Priority Metabolite Selection and Mendelian Randomization Instrument Construction

We leveraged summary-level metabolomic data from the UK Biobank (*N* = 274,241), quantified using the Nightingale Health nuclear magnetic resonance (NMR) platform, which measures 313 circulating metabolites and derived ratios [5]. Following the analytical framework of You et al. [5] and Qiang et al. [8], we identified 57 priority metabolites with genome-wide significant genetic instruments for downstream Mendelian randomization (MR) analyses. Priority metabolites were distributed across three tiers based on their predictive ranking for neurological disease endpoints: Tier 1 (ranked 1–5 in at least one disease; *n* = 18), Tier 2 (ranked 6–15; *n* = 19), and Tier 3 (ranked 16–30; *n* = 20).

For each of the 57 metabolites, we constructed MR instruments from genome-wide significant single-nucleotide polymorphisms (SNPs; *p* < 5 × 10^−8) with a minor allele frequency > 0.01. Instruments were pruned for linkage disequilibrium using a distance-based approach with a 10 Mb clumping window. Genomic coordinates were harmonized from GRCh37 (hg19) to GRCh38 (hg38) using liftOver (https://genome.ucsc.edu/cgi-bin/hgLiftOver, accessed on 1 May 2026), achieving a 99.96% success rate (1 SNP lost). The final instrument registry comprised 2472 genome-wide significant SNPs across 57 metabolites (Supplementary Table S1), with a mean F-statistic of 162.4 (range: 12–980+), indicating uniformly strong instruments that are well above the conventional threshold of F > 10 [13].

### 2.3. Cross-Disease Signature Discovery

We evaluated the metabolomic landscape across eight neurological disease endpoints from FinnGen and published GWAS consortia: Parkinson’s disease (PD; ieu-b-7 [9]), PD strict definition (PDSTRICT_EXMORE), Alzheimer’s disease (AD; ebi-a-GCST90027158 [10]), Alzheimer-type dementia (F5_ALZHDEMENT), vascular dementia (VasDem; finn-b-F5_VASCDEM), amyotrophic lateral sclerosis (ALS; ebi-a-GCST005647 [11]), multiple sclerosis (MS; ieu-b-18 [12]), and epilepsy (G6_EPLEPSY) as a neurological comparator. Epilepsy was included to test whether the pattern was restricted to progressive neurodegenerative proteinopathies or if it also appeared in a neurologically distinct endpoint. We therefore treated epilepsy as a specificity probe within the neurological spectrum, not as an independent validation of the signature. Quantitative overlap between the epilepsy and neurodegenerative disease top-30 metabolite sets is reported in Supplementary Section File S2 and Section 3.9. Frontotemporal dementia (FTD) and Lewy body dementia (LBD) were excluded due to insufficient case counts (N < 150 each) in the UK Biobank, which would preclude reliable metabolomic profiling.

For each metabolite–disease pair, we obtained the predictive rank from the metabolome-wide association analyses of You et al. [5] and Qiang et al. [8]. The predictive rank orders the priority metabolites within each disease by the strength of their baseline metabolite-to-incident-disease association (smaller rank = stronger association); rank 1 denotes the top-associated metabolite for that endpoint, and metabolites outside the top 30 are treated as unranked for that disease. Thus, the analysis evaluates pre-diagnostic prediction of future incident disease rather than contemporaneous diagnostic separation between established cases and controls. This distinction is important because the present design can identify circulating metabolites that precede diagnosis but cannot determine whether they are neurologically specific, causal, or downstream/prodromal markers. We then constructed a 57 × 8 metabolite–disease matrix, where each cell contained the rank of that metabolite for the corresponding disease (cells were left empty if the metabolite did not appear in the top 30 for that disease).

To identify a shared cross-neurological pre-diagnostic signature, we applied a coverage threshold: metabolites appearing in the top 30 for at least 7 of the 8 disease endpoints were designated as cross-disease signature members. This threshold (≥7 of 8 endpoints) was pre-specified in the analysis plan prior to results tabulation, based on repeated predictive ranking across the majority of endpoints examined. The top-30 cutoff is intentionally broad at the current instrument density (30/57, approximately 53% of the priority-metabolite set), so the permutation test should be interpreted as testing convergence under this specified ranking framework rather than as a stringent test of discriminant specificity. We did not construct a quantitative non-neurological disease control column because the complete disease-by-metabolite ranking matrix required for that analysis was not available for bulk download from the published resources or in the inspected local UK Biobank covariate export; consequently, specificity beyond the neurological endpoints is addressed qualitatively and as a limitation rather than through new quantitative claims. To assess threshold sensitivity, we conducted a post hoc 3 × 3 sensitivity analysis varying the rank cutoff (top 25, top 30, top 40) and disease coverage threshold (≥6, ≥7, ≥8 of 8 endpoints); results are reported in Supplementary Table S6. Bootstrap stability of signature membership was also assessed (1000 iterations; Supplementary Table S7). The significance of the observed cross-disease overlap was assessed via permutation testing (see Section 2.4).

We further classified the signature metabolites into interpretive biochemical categories based on their biochemical function: energy-related markers, ketone-related markers, and amino acid-/redox-related markers. This classification was performed independently of the statistical results and was based on established metabolic pathway annotations from the Human Metabolome Database (HMDB) and KEGG [14,15].

### 2.4. Permutation Testing

To assess whether the observed number of cross-disease signature metabolites exceeded chance expectation, we performed a permutation null test with 10,000 iterations (random seed = 42). The pre-specified coverage threshold was ≥7 of the 8 disease endpoints, applied across all eight neurological endpoints including epilepsy (see Section 2.3). For each permutation iteration, we independently reassigned which metabolites received a top-30 ranking within each disease column, preserving the total number of ranked metabolites per disease while randomizing metabolite identity. The permutation *p*-value was computed as the proportion of null iterations yielding a count equal to or exceeding the observed count. When no null iteration exceeded the observed count, *p*-values were reported as *p* < 1/n_iter_ (*p* < 0.0001 for *n* = 10,000), following the recommendation of Phipson and Smyth [16] to avoid reporting permutation *p*-values of exactly zero.

### 2.5. Mendelian Randomization Analyses

Mendelian randomization analyses were performed using the TwoSampleMR (0.7.4) R (4.6.0) package [17] with IVW as the primary estimator, supplemented by MR-Egger [18], weighted median [19], and MR-PRESSO (1.0) [20] sensitivity analyses. Steiger filtering [21] was applied to confirm directionality. For metabolite ratios, instruments were derived from the GWAS of the ratio variable directly (see Supplementary Methods File S1). Forward MR tested 45 metabolite–disease pairs (9 signature × 5 primary neurological disease outcomes) with Bonferroni correction for multiple testing (threshold: *p* < 0.00111).

### 2.6. Neurological Metabolite Score

To synthesize the 9-signature metabolites into a single composite metric, we derived a Neurological Metabolite Score (NMS) using summary-level rank data. For each metabolite *i* and disease *j*, we computed an inverse-rank weight:

*w_ij_* = (31 − *r_ij_*)/*sum_k_*(31 − *r_kj_*)

where *r_i_*_j_ denotes the rank of metabolite *i* for disease *j* (range: 1–30; metabolites not in the top 30 receive weight 0), k indexes all 9-signature metabolites, and the denominator *sum_k_* ensures that per-disease weights sum to 1. For application to individual-level metabolite data, each metabolite is expressed as a population z-score, z_i_ = (X_i_ − mu_i_)/sigma_i_, where X_i_ is the measured plasma level of metabolite *i* in a given individual, mu_i_ is the mean level of metabolite *i* in the reference population, and sigma_i_ is the corresponding population standard deviation; z_i_, therefore, re-expresses each metabolite on a common population-standardized scale (mean 0, standard deviation 1).

A disease-agnostic NMS weight for each metabolite was derived by averaging per-disease weights across only the diseases in which that metabolite received a non-zero weight (presence-weighted mean), then re-normalizing to sum to 1. This approach avoids systematically underweighting metabolites with 7/8 disease coverage relative to those with 8/8 coverage. The disease-agnostic NMS was computed as:

*NMS* = 0.148·*z*(*Creatinine*) + 0.139·*z*(*Acetone*) + 0.134·*z*(*Acetoacetate*)

+ 0.118·*z*(*Glucose*) + 0.115·*z*(*Ile*/*Leu*) + 0.102·*z*(*Lactate*/*Pyruvate*)

+ 0.097·*z*(*Gln*/*His*) + 0.075·*z*(*Citrate*/*Glucose*) + 0.072·*z*(*Lactate*)

The disease-agnostic NMS was applied conceptually as a weighted sum of population-standardized metabolite values, with z_i_ defined above. Per-disease weight column sums were verified to equal 1.000 for all 8 endpoints. Higher NMS values indicate a metabolomic profile more consistent with the shared systemic neurological signature described here.

The NMS is derived entirely from the same summary-level ranking framework used to define the 9-metabolite signature. It does not require individual-level UK Biobank access, but this portability should not be confused with validation. Application in an independent cohort with measured Nightingale NMR metabolites would constitute a future validation step, not an analysis completed here.

We emphasize that the NMS is a hypothesis-generating construct derived from the same rank data that identified the signature, creating circularity by design. It has not undergone external validation, held-out validation, AUC estimation, calibration testing, or clinical utility assessment. Because the NMS weights and signature definition are same-source constructs, leave-one-disease-out stability should be interpreted only as internal robustness evidence; it does not resolve circularity and does not substitute for external cohort validation. The score should be used only as a transparent summary of the rank-derived signature until independently validated.

## 3. Results

### 3.1. Fifty-Seven Priority Metabolites and 2472 Genetic Instruments

From the Nightingale NMR metabolomic platform applied to 274,241 UK Biobank participants (median follow-up: 14.9 years), we identified 57 priority metabolites with sufficient genome-wide significant genetic instruments for Mendelian randomization analyses across eight neurological disease endpoints (seven non-epilepsy neurological endpoints plus epilepsy as a comparator). The 57 metabolites spanned diverse biochemical categories, including amino acids, lipids, carbohydrates, ketone bodies, and derived metabolite ratios (Table S1).

The instrument registry comprised 2472 SNPs (mean 43.4 SNPs per metabolite; range: 7–82). All instruments exceeded the conventional strength threshold, with a mean F-statistic of 162.4 (range: 12–980+) and a mean minor allele frequency of 0.261. Tier 1 metabolites (ranked in the top five for at least one disease; *n* = 18) included established neurodegeneration-associated analytes such as glucose, creatinine, and acetoacetate, alongside less-characterized ratio markers. Tier 2 (*n* = 19) and Tier 3 (*n* = 20) metabolites exhibited progressively lower peak rankings but maintained consistent cross-disease representation.

**Figure 2:**
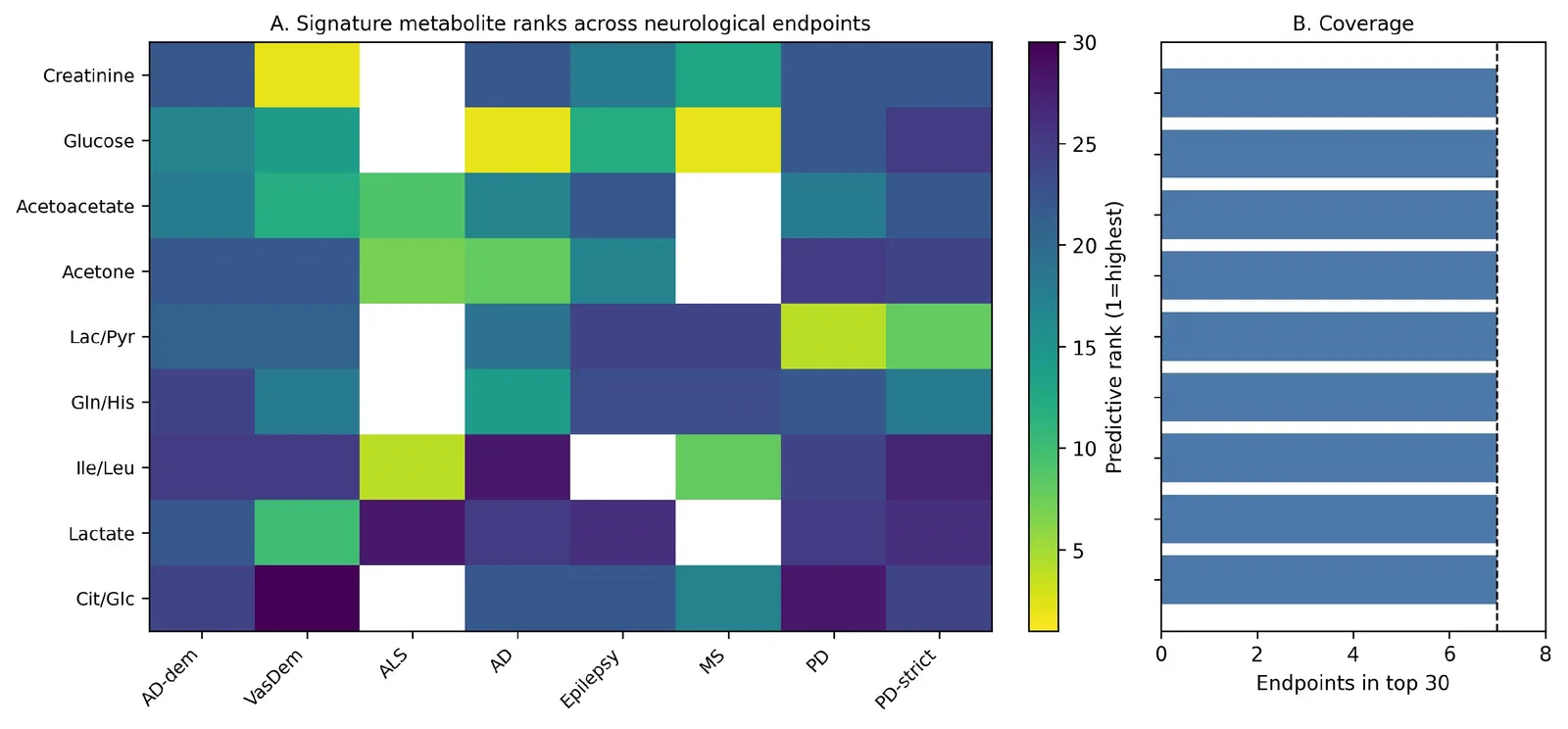
Cross-disease metabolomic landscape and the 9-metabolite shared systemic signature. (**A**) Heatmap of the 57 × 8 metabolite–disease-ranking matrix, showing the predictive rank of each priority metabolite for each neurological disease endpoint. Color intensity reflects rank position (darker = higher rank; gray = not ranked in top 30). (**B**) Horizontal bar chart identifying the 9 metabolites that appeared in the top 30 for at least 7 of 8 disease endpoints, constituting the shared systemic signature. Bars indicate the number of disease endpoints in which each metabolite was ranked in the top 30; the horizontal dashed line denotes the pre-specified ≥7/8 coverage threshold. The interpretive category schematic, empirical hierarchical clustering, and biochemical-category schematic are provided in Supplementary Figure S1.

The metabolite–disease-ranking landscape revealed substantial heterogeneity across disease endpoints (Figure 2A). Epilepsy, included as a neurological comparator (Section 2.3), displayed a ranking pattern that shared notable overlap with the neurodegenerative conditions (see Section 3.9 for detailed analysis). The two Parkinson’s disease definitions (PD and PD-strict) and the two Alzheimer’s definitions (AD and AD-dementia) showed highly correlated ranking profiles, providing internal consistency.

### 3.2. A Nine-Metabolite Shared Systemic Signature Across Neurological Endpoints

Applying the pre-specified coverage threshold of appearing in the top 30 for at least seven of the eight disease endpoints, we identified a signature of nine metabolites that repeatedly ranked among the baseline predictors of future incident neurological disease (Table 1; Figure 2B). The eight endpoints include two pairs of near-duplicate definitions (AD/AD-dementia, r = 0.883; PD/PD-strict, r = 0.933), so the effective number of independent endpoints is closer to six; the ≥7/8 coverage threshold and permutation-based convergence should be interpreted with this non-independence in mind. These nine metabolites were:

Table 1 lists each signature metabolite together with its biochemical category, best rank across the eight endpoints, and the single endpoint from which it was absent. Detailed biochemical interpretation of these metabolites is provided in the Discussion (Section 4.2), and the interpretive brain-level annotations referenced there are not directly indexed by the plasma measurements used here.

For descriptive readability, we grouped the nine-signature metabolites into three interpretive biochemical categories (Supplementary Figure S1A). The energy-related category (glucose, lactate, and the citrate/glucose ratio) captures glycolysis-to-TCA-cycle markers in plasma, while central glucose-processing abnormalities have also been reported in neurodegenerative disease using FDG-PET [6]. The ketone-related category (acetoacetate, acetone) reflects circulating ketogenesis and possible systemic fuel-shift physiology. The amino acid/redox-related category (creatinine, lactate/pyruvate ratio, glutamine/histidine ratio, and isoleucine/leucine ratio) collects markers related to redox balance, amino acid metabolism, body composition, and renal clearance. These groupings are interpretive aids, not data-derived mechanistic classes.

The nine-signature was not dominated by a single biochemical class, comprising five simple metabolites (creatinine, glucose, lactate, acetoacetate, and acetone) and four metabolite ratios (lactate/pyruvate, citrate/glucose, glutamine/histidine, and isoleucine/leucine). Metabolite ratios can capture relationships between linked biochemical quantities and have been proposed as informative features in genome-wide and metabolome-wide association analyses [22]. In this study, ratio members are interpreted as predictive summary markers rather than direct flux measurements.

The statistical significance of this cross-disease convergence was assessed via permutation testing (*p* < 0.0001; see Section 3.3). This test evaluates whether a repeated top-30 occurrence across the eight neurological endpoints exceeds random expectation under the specified ranking framework; it does not establish neurological specificity relative to non-neurological disease or distinguish causal from downstream/prodromal associations.

To assess whether the three interpretive biochemical categories correspond to a data-driven structure in the nine-signature metabolites, we performed hierarchical clustering of the nine metabolites based on Spearman’s rank correlation distance across all eight disease endpoints (Ward linkage; Supplementary Figure S1B). A three-cluster solution was selected only to compare with the number of interpretive categories. The data-driven clusters showed partial but incomplete correspondence with the prior category classification: metabolites from the same interpretive category did not consistently co-cluster, and two clusters each contained members from multiple categories. Ile/Leu formed a singleton cluster, consistent with its distinct absence pattern (missing only in epilepsy, while all other signature members are absent in either ALS or MS). These results indicate that the three-category classification is an interpretive biochemical framework rather than a strictly data-derived grouping, and that Supplementary Figure S1B provides the primary empirical rank-clustering structure.

### 3.3. Statistical Significance of Cross-Disease Convergence

**Figure 3:**
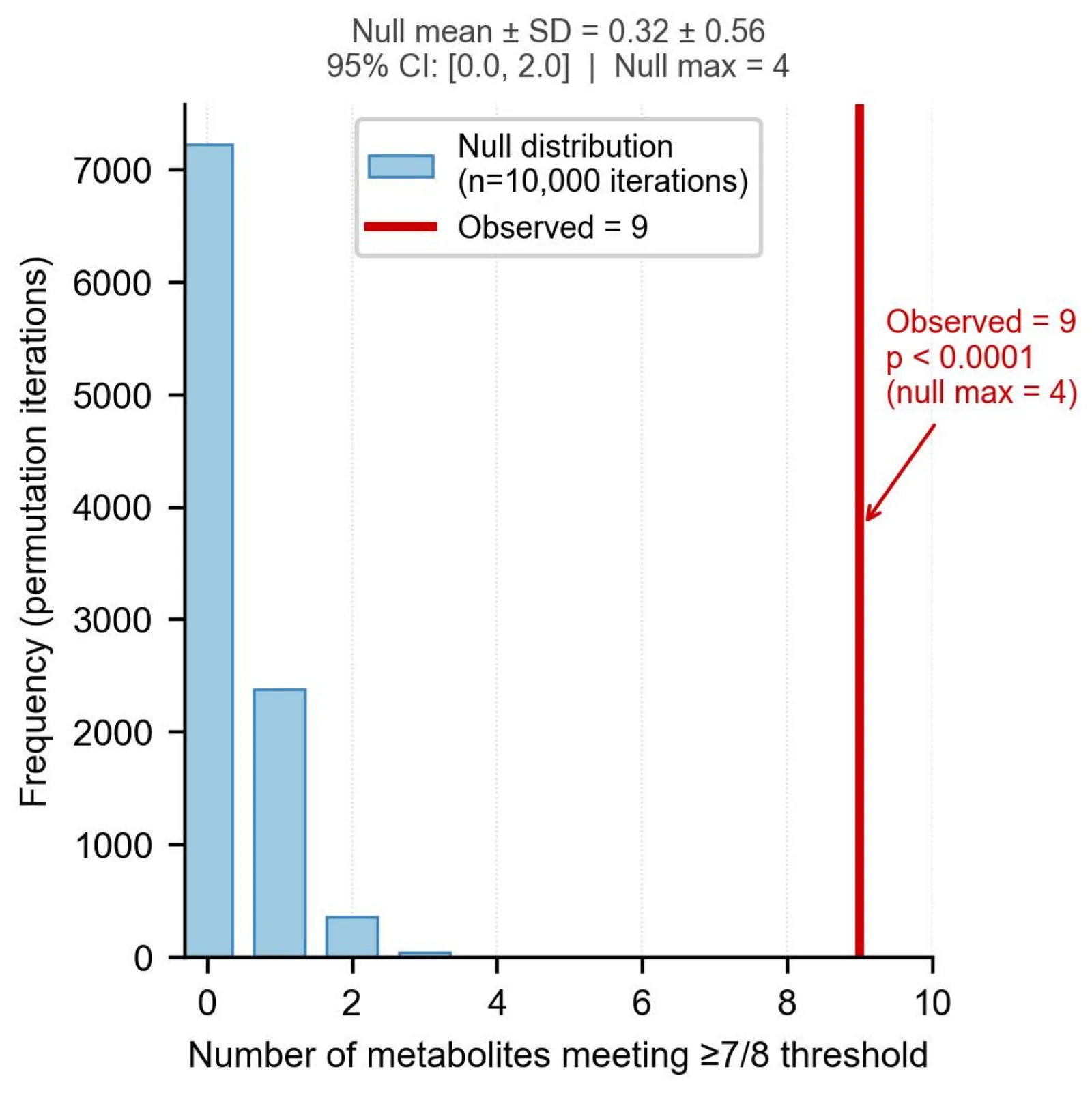
Permutation null distribution for cross-disease metabolite convergence. Histogram of the number of metabolites meeting the pre-specified ≥7/8 disease coverage threshold across 10,000 permutation iterations (random seed = 42). In each iteration, top-30 rankings were independently reassigned within each disease column while preserving the total number of metabolites ranked per disease. The observed count of 9 metabolites (red dashed vertical line) significantly exceeded the null distribution (null mean ± SD: 0.32 ± 0.56; null maximum = 4; permutation *p* < 0.0001), supporting non-random convergence across the analyzed neurological endpoints. This test does not establish neurological specificity relative to non-neurological disease.

To assess whether the cross-disease convergence of the nine-metabolite signature exceeded chance expectation, we performed a permutation null test (*n* = 10,000 iterations, seed = 42; Section 2.4). The observed count of nine metabolites meeting the ≥7/8 threshold significantly exceeded the null distribution (null mean ± SD: 0.32 ± 0.56; null 95% CI: [0.0, 2.0]; permutation *p* < 0.0001 [16]; Figure 3). No null permutation produced a count equal to or exceeding the observed value of 9 (null maximum = 4 across 10,000 iterations). These results support non-random convergence across the analyzed neurological endpoints, but they should not be read as evidence that the signature is specific to neurological disease or absent from other systemic morbidities.

### 3.4. Descriptive Biochemical Grouping of the Shared Signature

For readability, the nine-signature metabolites were summarized into three interpretive biochemical categories: energy-related markers, ketone-related markers, and amino acid/redox-related markers (Supplementary Figure S1C). These categories are descriptive and pathway-informed; the empirical rank-structure is shown in Supplementary Figure S1B, and the metabolite-level composition is provided in Table 1. Detailed mechanistic interpretation is reserved for the Section 4.

### 3.5. Forward Mendelian Randomization

**Figure 4:**
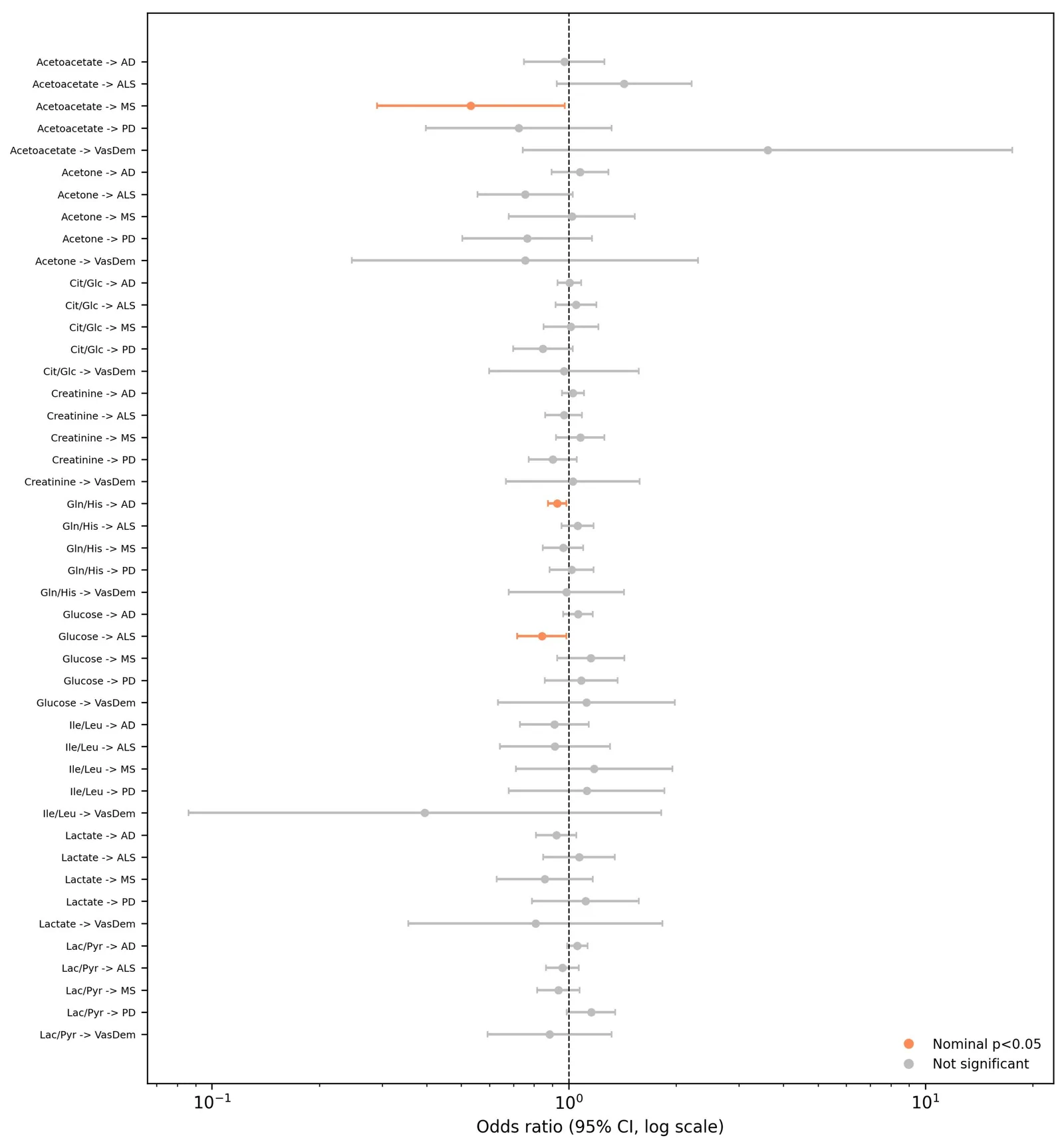
Forest plot of forward Mendelian randomization results for the 9-signature metabolites across 5 primary neurological disease outcomes. Each row represents one metabolite–disease pair (45 total: 9 metabolites × 5 diseases: PD, AD, ALS, MS, and vascular dementia). Circles indicate inverse variance-weighted (IVW) odds ratios; horizontal lines denote 95% confidence intervals. Three nominally significant pairs (*p* < 0.05) that did not survive Bonferroni correction (threshold: *p* < 0.00111 for 45 tests) are highlighted: glucose to ALS (OR = 0.841 [0.717–0.986], *p* = 0.033), acetoacetate to MS (OR = 0.532 [0.290–0.975], *p* = 0.041), and glutamine/histidine to AD (OR = 0.928 [0.874–0.986], *p* = 0.017). All three nominal associations are in the protective direction (OR < 1). The vertical dashed line at OR = 1.0 denotes the null hypothesis of no causal effect.

Forward Mendelian randomization analyses were performed for all 45 metabolite–disease pairs (nine signature metabolites × five primary neurological disease outcomes: PD, AD, ALS, MS, and vascular dementia), using genome-wide significant genetic instruments from the Nightingale NMR metabolite GWAS (range: 4–68 SNPs per pair after harmonization; Section 2.5). After Bonferroni correction for 45 tests (threshold: *p* < 0.00111), no metabolite–disease pair reached statistical significance. Three pairs showed nominal associations (*p* < 0.05) that did not survive multiple testing correction (Supplementary Table S3; Figure 4): higher genetically instrumented glucose levels were associated with a lower ALS risk (IVW OR = 0.841 [95% CI: 0.717–0.986], *p* = 0.033); higher acetoacetate levels were associated with a lower MS risk (OR = 0.532 [0.290–0.975], *p* = 0.041); and a higher glutamine/histidine ratio was associated with a lower AD risk (OR = 0.928 [0.874–0.986], *p* = 0.017). Given 45 tests, approximately 2.25 nominal findings would be expected at *p* < 0.05 under the global null, so these three nominal associations are treated as exploratory and not as causal support. Creatinine—the highest-weighted NMS component (w = 0.148)—showed no forward causal associations with any of the five disease endpoints (all *p* > 0.19), consistent with its role as a systemic state marker rather than as a direct causal upstream driver of neurological disease.

Complete IVW, MR-Egger, and weighted median results for all 45 pairs are reported in Supplementary Table S3. Interpretation of statistical power and the null causal evidence is provided in Section 4.3.

### 3.6. The Neurological Metabolite Score

Using inverse-rank weighting derived from the metabolite–disease-ranking matrix (Section 2.6), we constructed a disease-agnostic NMS as a weighted composite of the nine-signature metabolites (Table S2). The top-weighted metabolites were creatinine (w = 0.148), acetone (w = 0.139), and acetoacetate (w = 0.134). These weights are reported as a transparent rank-derived construct, with internal leave-one-disease-out robustness reported in Supplementary Tables S4 and S5; they should not be interpreted as externally validated prediction weights.

**Figure 5:**
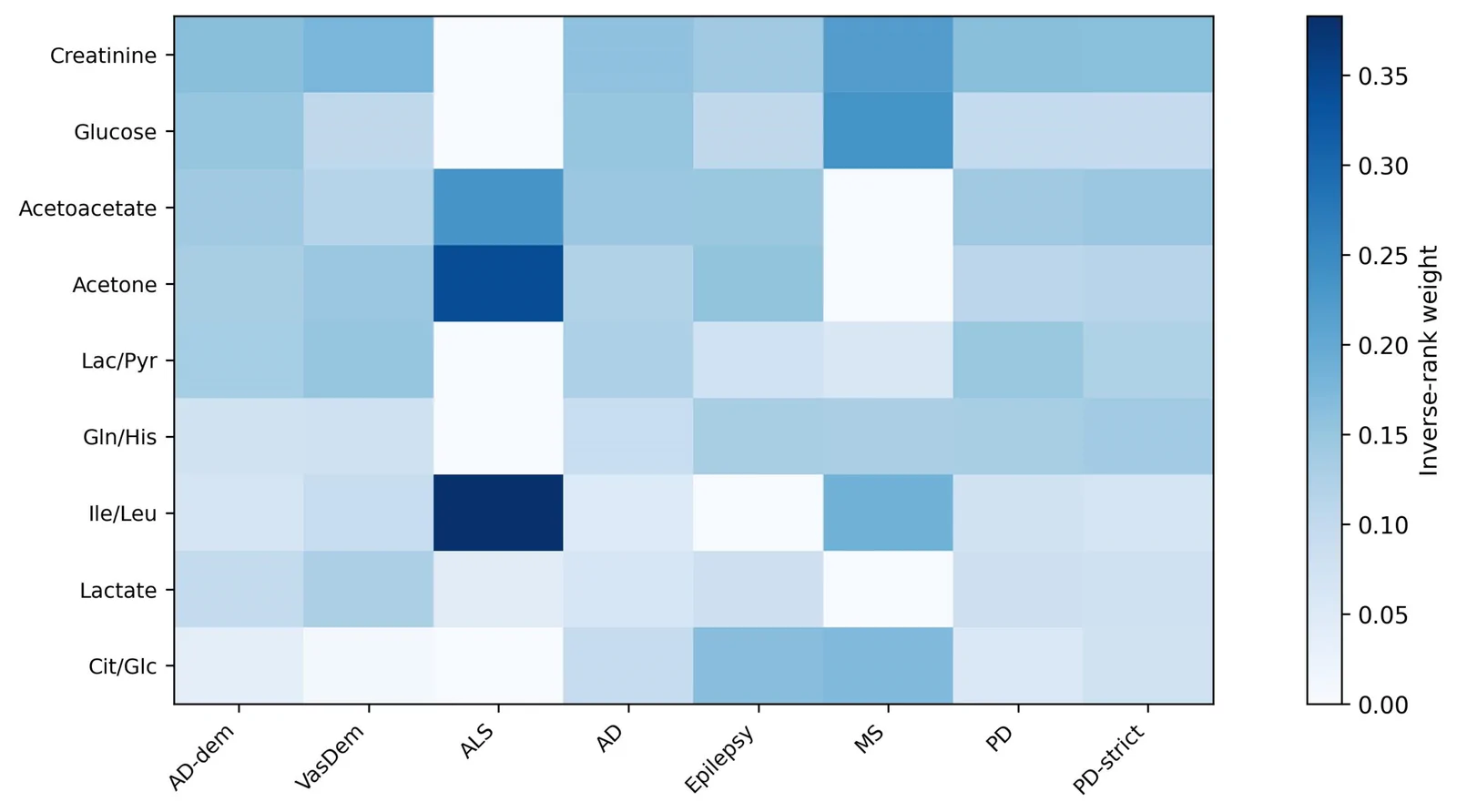
Disease-specific weight profiles of the Neurological Metabolite Score (NMS). Heatmap displaying the inverse-rank weights assigned to each of the 9-signature metabolites for each of the 8 neurological disease endpoints. Color intensity represents weight magnitude (darker = higher weight; white = absent from top 30 for that disease). The top-weighted metabolites overall are creatinine (disease-agnostic weight w = 0.148), acetone (w = 0.139), and acetoacetate (w = 0.134). These weights are shown as a transparent rank-derived construct, not as a validated prediction model. Creatinine should be interpreted primarily as a systemic body composition and renal-frailty proxy in this plasma analysis. Column sums equal 1.000 for all 8 disease endpoints.

Disease-specific weight profiles are shown in Figure 5 for transparency, but they should be interpreted descriptively. Creatinine’s high overall weight is most consistent with systemic body composition and renal-frailty physiology in this plasma analysis. Ketone body weights and citrate/glucose patterns may reflect systemic metabolic state, nutrition, or disease-proximal physiology; they should not be interpreted as proof of disease-specific brain mechanisms.

### 3.7. Internal Consistency of Signature Membership

To assess internal consistency of the nine-metabolite signature prior to external validation, we conducted analyses focused on signature membership, rank patterns, and NMS weight sensitivity. These analyses do not validate the NMS as a predictive score, but they test whether the rank-derived signature and weights are dominated by any single disease endpoint.

First, rank correlation stability across paired disease definitions confirmed high concordance: PD versus PD-strict (Spearman *r* = 0.773, *p* = 4.65 × 10^−18) and AD versus Alzheimer-type dementia (*r* = 0.880, *p* = 1.49 × 10^−28) across all 57 priority metabolites, indicating that the signature is robust to definitional variation within disease categories.

Second, mean pairwise rank correlation of the nine-signature metabolites across all neurological disease pairs was r = 0.258 (Supplementary Table S8), reflecting substantial heterogeneity across disease pairs—from strong concordance between AD-related definitions (AD vs. AD-dementia, r = 0.883) and PD definitions (PD vs. PD-strict, r = 0.933) to a modest negative correlation for ALS versus AD (r = −0.456) and ALS versus MS (r = −0.446). This heterogeneity underscores that the nine-signature metabolites are not uniformly co-ranked across all diseases but meet the coverage threshold despite between-disease differences.

Third, leave-one-disease-out analysis demonstrated complete stability of signature membership: when each of the eight disease endpoints was systematically excluded and the remaining metabolites were evaluated against a ≥6-of-7 threshold, all nine signature metabolites retained qualification in all eight exclusion scenarios. We performed a leave-one-endpoint-out analysis of the NMS weights using the per-disease weight matrix and the presence-weighted mean defined in Section 2.6. The full disease-agnostic weights were first reconstructed and matched the deposited disease-agnostic weights to numerical precision (maximum absolute difference = 1.39 × 10^−16). After excluding each endpoint in turn, NMS weight rankings remained highly concordant with the full weights (Spearman rho range: 0.883–1.000; Kendall tau range: 0.722–1.000). Metabolite-specific leave-one-disease-out weight ranges are reported in Supplementary Tables S4 and S5.

These analyses support the internal robustness of signature memberships and indicate that the NMS weight ranking is not driven by a single disease endpoint within the same UK Biobank-derived ranking framework. This does not resolve circularity because the NMS weights and signature definition remain derived from the same source rankings, and it does not replace external validation. Predictive performance, calibration, clinical utility, and transportability remain unassessed and require independent prospective cohorts with measured Nightingale NMR metabolites.

### 3.8. Threshold Sensitivity and Bootstrap Signature Stability

To evaluate whether the nine-metabolite signature is an artifact of the specific rank and coverage thresholds chosen, we conducted two complementary sensitivity analyses.

Threshold sensitivity (3 × 3 matrix): We repeated the signature discovery procedure across nine combinations of rank cutoff (top 25, top 30, top 40) and disease coverage threshold (≥6, ≥7, ≥8 of 8 endpoints). Across all nine combinations, the full nine-metabolite signature was retained (Core_9 = 9/9 in all combinations), and the three interpretive biochemical categories were preserved at all nine threshold combinations (Supplementary Table S6). The total number of qualifying metabolites varied across thresholds, as expected, supporting transparent sensitivity reporting rather than treating one cutoff as definitive.

Bootstrap stability: To assess robustness to variation in the metabolite panel, we performed 1000 bootstrap resamplings of the 57 metabolite rows. In each iteration, we re-applied the top-30/≥7-of-8 threshold and recorded which metabolites qualified. The nine pre-specified signature metabolites achieved mean bootstrap inclusion rates of 1.000 (range: 0.967 [Acetone]–1.024 [Glucose]; Supplementary Table S7). Bootstrap inclusion rates exceeding 1.0 reflect the with-replacement resampling design—each iteration samples with replacement, so a metabolite may contribute to multiple sampling positions—and indicate that all nine members are consistently rediscovered across panel perturbations. By comparison, the highest-inclusion non-signature metabolite (Gln_by_Gly, inclusion rate = 1.049) exceeded that of the lowest-inclusion signature member (Acetone, 0.967), indicating some overlap at the signature boundary that future studies with larger metabolite panels should evaluate.

### 3.9. Epilepsy as a Neurological Comparator: Specificity Probe

To assess the specificity of the nine-metabolite signature across neurological disease mechanisms, we examined the overlap between the epilepsy top-30 metabolite set and the other neurological disease top-30 sets (Supplementary Section File S2).

The epilepsy top-30 metabolite set overlapped substantially with those of the other neurological disease endpoints, with a mean overlap of 16.9 of 30 metabolites (56%), ranging from 7/30 (23%) for ALS to 22/30 (73%) for Alzheimer-type dementia. Of the nine shared signature metabolites, 8/9 appeared in the epilepsy top-30 set; the sole absent member was Ile/Leu—the only signature metabolite also absent from the epilepsy ranking, which is consistent with its distinct absence pattern relative to the other eight members. Because epilepsy was itself one of the eight endpoints used to define the signature (coverage threshold ≥ 7/8), this 8/9 overlap is, in part, expected by construction and should not be interpreted as independent validation; the disease-agnostic overlap statistic above (mean 16.9/30 across all top-30 sets, with epilepsy and ALS as low as 7/30) provides the more informative, non-circular measure of cross-disease convergence.

This finding indicates that the nine-metabolite signature is not restricted to progressive proteinopathies but also appears in epilepsy, a neurologically distinct endpoint characterized by episodic energy demand and responsiveness to ketogenic dietary interventions [23]. Rather than strengthening neurodegeneration specificity, the epilepsy overlap argues for a broader and less specific interpretation: the signature may index shared systemic and CNS metabolic stress across different neurological contexts.

These findings reframe the NMS from a neurodegenerative-specific risk index to a hypothesis-generating summary of a shared systemic neurological metabolic signature, warranting prospective validation across a broader spectrum of neurological conditions beyond the five primary neurological disease outcomes examined by forward MR here (see Section 4.6).

## 4. Discussion

### 4.1. Summary of Key Findings

This study presents a summary-level cross-disease metabolomic analysis spanning the neurological disease spectrum. By examining 57 priority metabolites across eight neurological disease endpoints in 274,241 UK Biobank participants, we identified a nine-metabolite signature that consistently appeared among the top 30 baseline predictors of future incident disease for at least seven of the eight endpoints. This pre-diagnostic design is a central strength: the signal is not simply a contemporaneous contrast between established cases and controls, but a pattern observed before recorded diagnosis in the source analyses. At the same time, it does not prove neurological specificity. We did not perform a quantitative non-neurological disease control analysis, and plasma metabolites such as glucose, lactate, ketone bodies, creatinine, and amino acid ratios may also reflect systemic morbidity, body composition changes, renal clearance, fasting status, or frailty. The most defensible interpretation is therefore a shared systemic, pre-diagnostic metabolic signature associated with future neurological disease risk, not a disease-specific or causal mechanism. Permutation testing confirmed that the observed cross-endpoint convergence exceeded random expectation within the analyzed neurological endpoints (*p* < 0.0001; null maximum = 4 across 10,000 iterations), but this result establishes non-random convergence rather than discriminant specificity. The overlap with epilepsy further argues against a strictly neurodegenerative-proteinopathy-specific interpretation and supports broader, non-specific neurological and systemic stress-marker framing.

### 4.2. Systemic Metabolic Stress and Brain-Energy Hypotheses

The identification of three recurrent biochemical categories provides an organizing framework for the shared systemic signature. The energy-related markers (glucose, lactate, and the citrate/glucose ratio) capture circulating glycolysis- and TCA-cycle-related information. These plasma findings sit alongside central glucose hypometabolism, reported by FDG-PET in neurodegenerative disorders [6] and CSF studies, including an AD-focused meta-analysis evaluating lactate, pyruvate, and related energy-metabolism markers [24]; however, this study did not measure brain or CSF metabolites and cannot infer direct plasma-to-brain correspondence.

The ketone-related markers (acetoacetate and acetone) likely reflect systemic fuel-shift physiology. Plasma acetoacetate and acetone are compatible with an altered ketogenic state, fasting or nutritional status, and compensatory metabolism, but the present data do not identify the tissue source or establish that the associations reflect neuronal fuel use.

The amino acid-/redox-related markers are heterogeneous. Creatinine is influenced by muscle mass and renal clearance, supporting its interpretation as a systemic or renal-frailty proxy in this analysis. The lactate/pyruvate, glutamine/histidine, and isoleucine/leucine ratios can be mapped to redox and amino acid biology, but the rank-based associations should be treated as biomarker signals rather than localization-specific mechanistic evidence.

### 4.3. Causal Inference from Forward Mendelian Randomization

Forward Mendelian randomization analyses evaluated causal relationships between genetically instrumented signature metabolite levels and neurodegenerative disease risk across 45 metabolite–disease pairs. No pair reached statistical significance after Bonferroni correction (threshold: *p* < 0.00111 for 45 tests). Three nominally significant associations emerged—glucose to ALS (*OR* = 0.841 [0.717–0.986]); acetoacetate to MS (*OR* = 0.532 [0.290–0.975]); glutamine/histidine to AD (*OR* = 0.928 [0.874–0.986])—but did not survive multiple testing correction and require independent replication before causal interpretation is warranted.

This null result under strict multiple testing correction is important for interpretation. The genetic instruments provide no Bonferroni-significant evidence that higher circulating levels of these signature metabolites causally drive or protect against the neurological disease outcomes tested here. Several considerations may contribute to this pattern. First, the polygenic architecture of neurological diseases distributes disease liability across many loci with small individual effects, limiting the per-SNP disease variance explained. Second, plasma metabolite levels may reflect downstream or prodromal physiological states rather than upstream causal factors. Third, MR instruments for metabolite ratios may capture numerator and denominator biology simultaneously, complicating causal interpretation despite Steiger filtering and pleiotropy sensitivity analyses. Fourth, nonlinear or disease-stage-specific effects from lifelong genetically proxied metabolite differences will be required before any causal interpretation is warranted.

The three nominal protective associations should be interpreted conservatively. With 45 tests, approximately 2.25 nominal associations would be expected at *p* < 0.05 by chance alone under the global null, and none of the observed associations survived Bonferroni correction. Their shared protective direction is hypothesis-generating, but it should not be used as causal evidence for the observational signature. Larger metabolite GWASs, independent outcome datasets, and designs that can separate preclinical disease effects from lifelong genetically proxied metabolite differences will be required before any causal interpretation is warranted.

The null forward MR result for creatinine across all five endpoints (all *p* > 0.19) is consistent with a state-marker role rather than a causal driver role. Creatine biosynthesis, muscle mass, mitochondrial creatine kinase activity, and renal clearance jointly determine creatinine levels [25]—a composite that may not translate into a directional causal signal against any single neurological endpoint via IVW. Creatinine thus appears to function primarily as a systemic body composition and renal-frailty marker in this analysis, observable as part of a broader prodromal or downstream metabolic milieu rather than as a causal upstream driver of individual proteinopathies.

### 4.4. Comparison with Prior Work

Our findings build on several complementary lines of evidence. You et al. [5] mapped the UK Biobank plasma metabolome to 527 prevalent and 859 incident diseases and established that metabolite profiles predict disease risk comparably to established clinical biomarkers. Their disease-by-disease analysis, however, did not examine cross-disease metabolomic convergence. Buergel et al. [26] trained a neural network on 168 NMR metabolic markers in 117,981 UK Biobank participants to predict 24 common diseases simultaneously; their multi-disease prediction model demonstrated that metabolomic profiles carry disease-relevant information across organ systems, but the analysis evaluated predictive accuracy per disease rather than testing whether specific metabolites recur across disease rankings. Qiang et al. [8] characterized the genetic architecture of the plasma metabolome in 254,825 individuals, providing the genome-wide significant metabolite instruments that underlie our Mendelian randomization analyses. Our study bridges these foundational datasets by explicitly asking which metabolomic signals recur across the neurodegenerative spectrum—a question that none of these prior studies addressed.

As a hypothesis-generating interpretation, the observed plasma pattern is compatible with a bioenergetic crisis framework in which altered substrate availability, ketone metabolism, and redox-related physiology accompany the prodromal phase of neurological disease. This interpretation remains speculative because the present study used summary-level plasma rankings and did not test brain, CSF, tissue-specific, or longitudinal within-person metabolic changes.

Robinson et al. [3] documented extensive co-pathology of protein aggregates across neurodegenerative diseases, demonstrating that amyloid, tau, alpha-synuclein, and TDP-43 frequently co-occur within the same brain. Lian et al. [4] further showed that data-driven subtyping of neurodegenerative diseases reveals overlapping biological subtypes that transcend clinical diagnostic boundaries. Our metabolomic signature provides a molecular counterpart to these neuropathological and genetic observations, suggesting that convergent metabolomic vulnerability may be an upstream factor contributing to the proteinopathy overlap documented at autopsy.

Our shared metabolomic signature coexists with disease-specific metabolomic information rather than replacing it. Prior studies have demonstrated that plasma and cerebrospinal fluid metabolomic profiling captures disease-specific metabolic alterations in Parkinson’s disease and Alzheimer’s disease, reflecting distinct downstream metabolic consequences of different proteinopathies [27,28]. The nine signature metabolites represent a shared systemic layer, while the remaining 48 priority metabolites may carry disease-discriminatory information. This two-tier architecture parallels the neuropathological finding that shared and disease-specific protein aggregates coexist in the same brain [3].

Berezhnoy et al. [7] demonstrated that NMR metabolomic profiling can stratify Parkinson’s disease, but their analysis was limited to a single disease. In our own prior work, machine learning analysis of targeted plasma proteomics predicted motor progression in Parkinson’s disease, underscoring the value of blood-based molecular markers for individualized neurodegenerative prognosis while remaining confined to a single disease [29]. The disease-agnostic Neurological Metabolite Score (NMS) proposed here extends this stratification concept across multiple neurological endpoints only as a transparent rank-derived construct; it has no AUC, calibration estimate, held-out validation, or external validation in the present study.

### 4.5. Limitations

Several limitations warrant acknowledgment. First, the UK Biobank cohort, while providing exceptional scale, is predominantly of European ancestry (>94%), limiting the generalizability of our findings to other populations. Metabolomic profiles may differ across ancestral groups due to genetic, dietary, and environmental factors, and, as such, replication in diverse cohorts is essential. Second, frontotemporal dementia (FTD) and Lewy body dementia (LBD) were excluded due to insufficient case counts in the UK Biobank (N < 150 each), precluding reliable metabolomic profiling. Third, the Nightingale NMR platform covers a subset of the total plasma metabolome; mass spectrometry-based platforms may identify additional cross-disease signals not detectable by NMR. Fourth, the two source studies use the same UK Biobank Nightingale NMR platform but differ in analytic purpose: You et al. provide metabolite-phenome prediction rankings, whereas Qiang et al. provide metabolite GWAS instruments. Any unreported or non-identical covariate adjustment in the source models is inherited here. A local UK Biobank individual-level covariate export was available for inspection and confirmed that demographic, anthropometric, lifestyle, blood biochemistry, genetic QC/principal-component, medication/self-reported illness, and hospital-/death-record fields exist locally; however, it did not contain the complete Nightingale metabolomics panel or the disease-by-metabolite ranking matrix needed to re-run the source models. This export was intentionally not used for partial re-adjustment because doing so would combine a summary-level ranking analysis generated under the original complete-panel source models with a different and incomplete individual-level dataset, yielding non-comparable estimates rather than a valid correction for confounding. If source models did not fully adjust for eGFR or renal function, BMI/body composition, fasting/sampling state, medication, nutrition, or lifestyle, the shared signature may partly reflect those systemic factors. Fifth, shared UK Biobank sampling and shared control structures across disease endpoints may inflate apparent cross-disease convergence relative to fully independent disease cohorts. In addition, the eight endpoints include two near-duplicate definition pairs (AD/AD-dementia, r = 0.883; PD/PD-strict, r = 0.933), making the effective number of independent endpoints closer to six; the ≥7/8 coverage threshold and permutation null should therefore be interpreted as evidence of convergence under this endpoint framework, not as eight fully independent replications. Sixth, our top-30 threshold is broad (30/57, approximately 53% of the priority metabolites), and several signature members are metabolite ratios whose numerator and denominator components are biologically correlated; permutation results should therefore be interpreted as evidence of non-random convergence under the specified framework, not as discriminant specificity. Seventh, we did not perform a direct case–control comparison of blood metabolomes between neurologically healthy individuals and diagnosed patients, nor a quantitative non-neurological disease control analysis because the complete disease-by-metabolite ranking matrix required for such contrasts was not available for reliable bulk extraction. The nine signature metabolites are abundant circulating compounds whose levels can change with a broad range of pathological and physiological states; in the absence of a healthy-versus-diseased diagnostic contrast, we cannot establish that they are qualitatively distinct from metabolites altered in general morbidity. The signature therefore cannot be considered neurologically specific and should be interpreted as a shared systemic, pre-diagnostic marker rather than as a neurologically specific diagnostic discriminator; it may partly reflect systemic morbidity, frailty, renal function, body composition, or nutritional states. Eighth, the ranking data derive from observational associations between baseline metabolite levels and incident disease diagnoses; prodromal disease processes may already influence metabolites before diagnosis. Ninth, the application of Mendelian randomization to metabolite ratios introduces ratio-specific methodological considerations; sensitivity analyses provide partial protection against directional pleiotropy but cannot fully resolve numerator–denominator biology.

### 4.6. Future Directions

The nine-metabolite shared systemic signature and the derived NMS open several avenues for future investigation. First, external validation of the NMS in independent prospective cohorts with complete Nightingale NMR data—including non-European populations and cohorts with explicit renal function, BMI/body composition, fasting, medication, and lifestyle covariates—is a priority. This emphasis on measurement fidelity and cross-dataset validation is consistent with our prior biomarker work across related neurological measurement settings [30,31]. Second, future analyses should include non-neurological disease comparators using a complete disease-by-metabolite ranking resource, if available, to quantify the degree of discriminant specificity. Third, the interpretive biochemical categories identified here can guide hypothesis testing around systemic energy substrate availability, ketone metabolism, amino acid balance, renal function, and frailty. Fourth, longitudinal metabolomic profiling may help distinguish stable pre-diagnostic risk markers from dynamic prodromal changes. Finally, threshold sensitivity and bootstrap stability should continue to be reported transparently; the present manuscript points to the existing 3 × 3 threshold analysis (Table S6) and bootstrap membership analysis (Table S7) rather than treating one threshold as definitive.

## 5. Conclusions

This study identifies a nine-metabolite shared systemic metabolic signature from 274,241 UK Biobank participants that recurs across baseline predictors of future incident neurological disease, including neurodegenerative, inflammatory demyelinating, vascular cognitive, and epileptic endpoints. The signature can be organized into three interpretive biochemical categories—energy-related, ketone-related, and amino acid-/redox-related markers, but these categories are not themselves proof of a single mechanism. The overlap with epilepsy indicates that the signature is not restricted to progressive proteinopathies and may index broader systemic or neurological metabolic stress. Forward Mendelian randomization provided no Bonferroni-significant evidence that the signature metabolites causally drive neurological disease outcomes, supporting a biomarker or prodromal-state interpretation. The summary-level NMS derived from this signature remains an unvalidated, hypothesis-generating construct with internal leave-one-disease-out robustness but no external validation; external prospective validation, held-out performance evaluation, and non-neurological comparators are essential before clinical or mechanistic claims are made.

## Figures and Tables

**Table 1:** Characteristics of the 9-metabolite shared systemic signature identified from 274,241 UK Biobank participants. Each metabolite appeared in the top 30 predictive metabolites for at least 7 of 8 neurological disease endpoints. Best rank indicates the highest rank achieved across all 8 endpoints. Absent from indicates the single endpoint where the metabolite did not appear in the top 30. Axis labels are retained as interpretive biochemical categories, not data-proven mechanisms. Mechanistic annotations refer to brain-level biochemistry where relevant; this study measured plasma metabolites, which do not directly index brain or CSF processes.

Axis	#	Metabolite	Category	Best Rank	Endpoints in Top 30	Absent From
Energy	1	Glucose	Carbohydrate	2	7/8	ALS
Energy	2	Lactate	Organic acid	10	7/8	MS
Energy	3	Citrate/Glucose	Metabolite ratio	1	7/8	ALS
Ketone	4	Acetoacetate	Ketone body	3	7/8	MS
Ketone	5	Acetone	Ketone body	3	7/8	MS
AA/Redox	6	Creatinine	Small molecule	1	7/8	ALS
AA/Redox	7	Lactate/Pyruvate ratio	Metabolite ratio	4	7/8	ALS
AA/Redox	8	Glutamine/Histidine ratio	Amino acid ratio	5	7/8	ALS
AA/Redox	9	Isoleucine/Leucine ratio	BCAA ratio	8	7/8	Epilepsy

## Data Availability

The UK Biobank metabolomic data used in this study are available from the UK Biobank upon application (http://www.ukbiobank.ac.u, accessed on 1 May 2026). The metabolite–disease ranking matrices and MR instrument registry are publicly available via Figshare (DOI: 10.6084/m9.figshare.29390471). The disease-agnostic Neurological Metabolite Score weight matrix is deposited on Zenodo (DOI: 10.5281/zenodo.19712955) and is publicly available. The full analysis code will be released in a public GitHub repository under the MIT license upon publication.

## Supplementary Materials

The following supporting information can be downloaded at: https://www.mdpi.com/article/10.3390/biomedicines14081773/s1, Table S1. Characteristics of the 57 priority metabolites with genome-wide significant genetic instruments. Table S2. Neurological Metabolite Score (NMS) per-disease and disease-agnostic weight matrix. Table S3. Complete forward Mendelian randomization results for the 9 signature metabolites × the 5 primary neurological disease outcomes. Table S4. NMS leave-one-endpoint-out weight-ranking concordance. Table S5. Metabolite-specific NMS weight ranges across the leave-one-endpoint-out runs. Table S6. Threshold sensitivity analysis: retention of the 9-metabolite signature across 9 combinations. Table S7. Bootstrap stability of 9-signature membership (n = 1000 iterations). Table S8. Internal consistency: Spearman rank correlations for the 9 signature metabolites across neurological disease pairs. Supplementary Methods File S1. Mendelian randomization for metabolite ratios. Supplementary Section File S2. Epilepsy overlap analysis. Figure S1. Interpretive category classification, empirical rank clustering, and biochemical-category schematic for the 9-metabolite shared systemic signature: (A) interpretive biochemical classification (energy-, ketone-, and amino acid/redox-related); (B) hierarchical clustering dendrogram (Spearman rank-correlation distance, Ward linkage) across all 8 neurological endpoints; (C) biochemical-category schematic (arrows denote hypotheses, not causal evidence).

## Abbreviations

**Abbreviation**	**Full term**
AD	Alzheimer’s disease
ALS	Amyotrophic lateral sclerosis
BCAA	Branched-chain amino acid
CI	Confidence interval
FDG-PET	Fluorodeoxyglucose positron emission tomography
FTD	Frontotemporal dementia
GWAS	Genome-wide association study
IVW	Inverse variance-weighted
LBD	Lewy body dementia
LD	Linkage disequilibrium
MAF	Minor allele frequency
MR	Mendelian randomization
MS	Multiple sclerosis
NMR	Nuclear magnetic resonance
NMS	Neurological Metabolite Score
OR	Odds ratio
PD	Parkinson’s disease
SNP	Single nucleotide polymorphism
TCA	Tricarboxylic acid (cycle)
UKB	UK Biobank
VasDem	Vascular dementia
WM	Weighted median
