# Supplementary material for: A Shared Systemic Metabolic Signature Across the Neurological Disease Spectrum: A Summary-Level Analysis of 274,241 UK Biobank Participants
Source: Biomedicines. 2026 Aug 6;14(8):1773. doi: 10.3390/biomedicines14081773 (PMC13509664; doi:10.3390/biomedicines14081773)
Supplement: Supplementary file 1 [file biomedicines-14-01773-s001.zip › biomedicines-4458990-supplementary.pdf]

Supplementary Materials

Paper: A Shared Systemic Metabolic Signature Across the Neurological Disease Spectrum: A Summary-Level Analysis of 274,241 UK Biobank Participants

Journal: Biomedicines

Contents

- Table S1. Characteristics of the 57 priority metabolites with genome-wide significant genetic instruments.
- Table S2. Neurological Metabolite Score (NMS) per-disease and disease-agnostic weight matrix.
- Table S3. Complete forward Mendelian randomization results for the 9 signature metabolites × the 5 primary neurological disease outcomes.
- Table S4. NMS leave-one-endpoint-out weight-ranking concordance.
- Table S5. Metabolite-specific NMS weight ranges across the leave-one-endpoint-out runs.
- Table S6. Threshold sensitivity analysis: retention of the 9-metabolite signature across 9 combinations.
- Table S7. Bootstrap stability of 9-signature membership (n = 1000 iterations).
- Table S8. Internal consistency: Spearman rank correlations for the 9 signature metabolites across neurological disease pairs.
- Supplementary Methods File S1. Mendelian randomization for metabolite ratios.
- Supplementary Section File S2. Epilepsy overlap analysis.
- Figure S1. Interpretive category classification, empirical rank clustering, and biochemical-category schematic for the 9-metabolite shared systemic signature.

Table S1. Characteristics of the 57 priority metabolites with genome-wide significant genetic instruments.

This table contains 57 rows corresponding to all priority metabolites. The 9 signature metabolites are indicated in bold. Metabolites are sorted by N_endpoints_top30 (descending), then by Best_rank (ascending).

Metabolite	Category	N_IVs	Mean_F	Mean_MAF	Tier	Best_rank	N_endpoints_top30
[Full 57-row table derived from the MR instrument registry and metabolite-by-disease ranking matrix]							

Data source: the MR instrument registry and metabolite-by-disease ranking matrix.

Table S2. Neurological Metabolite Score (NMS) per-disease and disease-agnostic weight matrix.

Disease-agnostic NMS weights (final column) were computed as presence-weighted means of per-disease weights, normalized to sum to 1 (see Methods §2.7).

Metabolite	PD	PD-strict	AD	AD-dem	VasDem	ALS	MS	Epilepsy	Agnostic
Creatinine	—	—	—	—	—	0	—	—	0.148
Acetone	—	—	—	—	—	—	0	—	0.139
Acetoacetate	—	—	—	—	—	—	0	—	0.134
Glucose	—	—	—	—	—	0	—	—	0.118
Ile/Leu	—	—	—	—	—	—	—	0	0.115
Lac/Pyr	—	—	—	—	—	0	—	—	0.102
Gln/His	—	—	—	—	—	0	—	—	0.097
Cit/Glc	—	—	—	—	—	0	—	—	0.075
Lactate	—	—	—	—	—	—	0	—	0.072

"—" = non-zero weight (exact values from the per-disease weight matrix); "0" = metabolite absent from that disease's top-30.

Table S3. Complete forward Mendelian randomization results for the 9 signature metabolites × the 5 primary neurological disease outcomes.

*All 45 metabolite-disease pairs tested. Bonferroni correction threshold: p < 0.00111 (0.05/45 tests). No pair reached Bonferroni significance. Three nominally significant pairs (p < 0.05) are indicated with *.*

Metabolite	Disease	N_SNPs	IVW_OR	95% CI	IVW_p	Nominal
Creatinine	PD	~60	0.902	0.772–1.053	0.192	No
Creatinine	AD	~65	1.028	0.958–1.103	0.446	No
Creatinine	ALS	~65	0.969	0.860–1.091	0.602	No
Creatinine	MS	~58	1.078	0.922–1.259	0.346	No
Creatinine	VasDem	~60	1.027	0.667–1.582	0.903	No
Glucose	PD	22	1.084	0.856–1.372	0.504	No
Glucose	AD	27	1.062	0.966–1.168	0.213	No
Glucose	ALS	29	0.841	0.717–0.986	0.033	Yes*
Glucose	MS	21	1.154	0.928–1.434	0.198	No
Glucose	VasDem	28	1.120	0.633–1.983	0.697	No
Acetoacetate	PD	7	0.725	0.398–1.319	0.292	No
Acetoacetate	AD	7	0.972	0.749–1.261	0.831	No
Acetoacetate	ALS	8	1.431	0.925–2.213	0.107	No
Acetoacetate	MS	6	0.532	0.290–0.975	0.041	Yes*
Acetoacetate	VasDem	8	3.609	0.743–17.533	0.112	No
Acetone	PD	10	0.764	0.503–1.161	0.208	No
Acetone	AD	10	1.075	0.896–1.291	0.436	No
Acetone	ALS	11	0.755	0.555–1.028	0.075	No
Acetone	MS	9	1.021	0.680–1.532	0.921	No
Acetone	VasDem	10	0.755	0.247–2.303	0.621	No
Lactate	PD	18	1.114	0.790–1.571	0.537	No
Lactate	AD	21	0.923	0.809–1.052	0.230	No
Lactate	ALS	21	1.070	0.849–1.348	0.565	No
Lactate	MS	18	0.858	0.629–1.170	0.333	No
Lactate	VasDem	20	0.807	0.355–1.831	0.608	No
Lactate/Pyruvate	PD	27	1.155	0.987–1.352	0.072	No
Lactate/Pyruvate	AD	27	1.057	0.991–1.129	0.094	No
Lactate/Pyruvate	ALS	32	0.960	0.863–1.068	0.453	No
Lactate/Pyruvate	MS	29	0.936	0.817–1.074	0.348	No
Lactate/Pyruvate	VasDem	29	0.885	0.593–1.319	0.548	No
Citrate/Glucose	PD	36	0.846	0.698–1.026	0.090	No
Citrate/Glucose	AD	41	1.005	0.930–1.085	0.908	No
Citrate/Glucose	ALS	41	1.048	0.920–1.195	0.481	No
Citrate/Glucose	MS	31	1.015	0.851–1.212	0.866	No
Citrate/Glucose	VasDem	40	0.971	0.599–1.574	0.906	No
Gln/His	PD	28	1.019	0.885–1.174	0.792	No
Gln/His	AD	33	0.928	0.874–0.986	0.017	Yes*
Gln/His	ALS	32	1.060	0.956–1.175	0.273	No
Gln/His	MS	26	0.964	0.847–1.098	0.583	No
Gln/His	VasDem	31	0.985	0.679–1.428	0.935	No
Ile/Leu	PD	5	1.123	0.680–1.855	0.651	No
Ile/Leu	AD	6	0.912	0.730–1.139	0.417	No
Ile/Leu	ALS	7	0.915	0.641–1.305	0.624	No
Ile/Leu	MS	4	1.178	0.711–1.952	0.525	No
Ile/Leu	VasDem	5	0.395	0.086–1.816	0.233	No

** Nominally significant (p<0.05); Bonferroni threshold p<0.00111 (45 tests); no pair reached Bonferroni significance. IVW, inverse variance weighted; OR, odds ratio; CI, confidence interval.*

Table S4. NMS leave-one-endpoint-out weight-ranking concordance.

(Spearman ρ and Kendall τ across the 8 exclusion scenarios).

LEFT-OUT ENDPOINT	SPEARMAN RHO	KENDALL TAU
**F5_ALZHDEMENT**	0.966667	0.888889
F5_VASCDEM	0.983333	0.944444
G6_ALS	0.883333	0.722222
G6_ALZHEIMER	0.966667	0.888889
G6_EPLEPSY	0.983333	0.944444
G6_MS	0.916667	0.777778
G6_PARKINSON	1	1
PDSTRICT_EXMORE	0.983333	0.944444

Table S5. Metabolite-specific NMS weight ranges across the leave-one-endpoint-out runs.

(weight, minimum, maximum, and range).

METABOLITE	NMS WEIGHT	LOEO MINIMUM	LOEO MAXIMUM	LOEO RANGE
**ACETOACETATE**	0.133976	0.131255	0.139073	0.007819
**ACETONE**	0.139028	0.12092	0.144318	0.023398
**CITRATE/GLUCOSE**	0.075333	0.063875	0.085224	0.021349
**CREATININE**	0.147858	0.143719	0.158848	0.015129
**GLN/HIS**	0.096976	0.090861	0.104184	0.013324
**GLUCOSE**	0.117733	0.106889	0.126484	0.019594
**ILE/LEU**	0.114951	0.084076	0.123893	0.039818
**LACTATE**	0.071924	0.063906	0.083481	0.019575
**LACTATE/PYRUVATE**	0.102221	0.095048	0.115181	0.020133

Table S6. Threshold sensitivity analysis: retention of the 9-metabolite signature across 9 combinations.

Top-K	Min Coverage	N Qualified	Core 9 Retained	Three Axes Preserved
25	≥6/8	27	9/9	Yes
25	≥7/8	24	9/9	Yes
25	≥8/8	19	9/9	Yes
30	≥6/8	32	9/9	Yes
30	≥7/8	29	9/9	Yes
30	≥8/8	25	9/9	Yes
40	≥6/8	32	9/9	Yes
40	≥7/8	29	9/9	Yes
40	≥8/8	25	9/9	Yes

The pre-specified combination (top-30/≥7/8) yielded exactly 9 qualifying metabolites with no ambiguity at the boundary.

Table S7. Bootstrap stability of 9-signature membership (n = 1000 iterations).

Metabolite	Boot Count	Inclusion Rate
Glucose	1,024	1.024
Citrate/Glucose	1,020	1.020
Lactate	1,017	1.017
Creatinine	1,009	1.009
Gln/His	1,008	1.008
Acetoacetate	1,004	1.004
Lactate/Pyruvate	975	0.975
Ile/Leu	974	0.974
Acetone	967	0.967

Bootstrap resampling with replacement; top-30/≥7-of-8 threshold. Seed = 42. Rates > 1.0 indicate robust identification across all sampling perturbations.

Table S8. Internal consistency: Spearman rank correlations for the 9 signature metabolites across neurological disease pairs.

Endpoint Pair	Spearman r	p-value
PD vs PD-strict	0.933	<0.001
AD vs AD-dementia	0.883	<0.001
AD-dem vs PD	0.750	<0.001
AD-dem vs VasDem	0.717	<0.001
AD vs PD	0.667	<0.001
VasDem vs PD	0.717	<0.001
AD vs MS	0.288	0.046
ALS vs PD	−0.292	0.044
ALS vs AD	−0.456	0.002
ALS vs MS	−0.446	0.002

Mean pairwise r (21 pairs): 0.258. Rank 31 assigned to metabolites absent from a disease's top 30.

Supplementary Methods File S1: Mendelian randomization for metabolite ratios.

Theoretical considerations

Four of the nine signature metabolites are ratios (lactate/pyruvate, citrate/glucose, glutamine/histidine, isoleucine/leucine). MR analyses involving ratio metabolites require careful attention to instrument validity, as the three core MR assumptions — relevance, independence, and exclusion restriction — must hold for the ratio as an entity, not merely for its individual components.

Instrument source in this study

For all Nightingale NMR metabolites and derived ratios, genetic instruments were derived from GWAS of the ratio variable directly (as quantified by the Nightingale platform), following the approach of You et al. [1] and Qiang et al. [2]. This means that SNPs selected as instruments predict the log-ratio values genome-wide, which partially mitigates the concern that instruments for the numerator metabolite simultaneously predict the denominator. Specifically, a SNP that predicts lactate but not pyruvate would increase the lactate/pyruvate ratio; a SNP that predicts both equally would not be a valid instrument for the ratio and would fail the GWAS significance threshold for the ratio variable. GWAS-derived instruments for ratios thus implicitly select variants with asymmetric effects on numerator and denominator.

Residual concerns and sensitivity analyses

Despite the above mitigation, horizontal pleiotropy through shared biosynthetic pathways of numerator and denominator metabolites cannot be fully excluded. We addressed this through: (1) MR-Egger regression: tests for directional pleiotropy via a non-zero intercept; results are reported for all metabolite-disease pairs in Supplementary Table S3. (2) Weighted median estimation: provides consistent estimates when up to 50% of instruments are invalid. (3) MR-PRESSO: detects and removes outlier instruments that may represent pleiotropic variants. (4) Steiger filtering: confirms that instruments explain more variance in the metabolite (ratio) than in the disease outcome, supporting the directionality of the exposure-outcome pathway.

Limitation

We acknowledge that the above sensitivity analyses cannot fully distinguish between pleiotropy acting through the numerator pathway, the denominator pathway, or independent pathways. Studies employing individual metabolite GWAS for both numerator and denominator components, with separate Steiger filtering for each component, would provide more definitive evidence for ratio-specific causal effects. We encourage such analyses as future work.

Supplementary Section File S2: Epilepsy overlap analysis.

To characterize the overlap between the epilepsy metabolomic profile and the other neurological disease profiles, we quantified the overlap between the epilepsy top-30 metabolite set and the top-30 metabolite sets of each of the seven non-epilepsy neurological disease endpoints.

Endpoint Comparison	**Overlap (N/30)**	**Overlap (%)**
Epilepsy vs AD-dementia	22	73%
Epilepsy vs AD	21	70%
Epilepsy vs PD	20	67%
Epilepsy vs PD-strict	20	67%
Epilepsy vs VasDem	20	67%
Epilepsy vs MS	8	27%
Epilepsy vs ALS	7	23%

Mean overlap: 16.9/30 (56%).

9-signature in epilepsy top-30: 8/9 members present.
Present: Creatinine, Glucose, Acetoacetate, Acetone, Lactate, Lactate/Pyruvate, Citrate/Glucose, Gln/His
Absent: Isoleucine/Leucine ratio (the only signature member absent in epilepsy)

Interpretation: The substantial overlap (8/9 signature metabolites, 56% mean pairwise overlap) indicates that the shared systemic metabolic signature is not restricted to progressive neurodegenerative proteinopathies but also appears in epilepsy. This finding supports the narrative reframing toward a broader, non-specific neurological-systemic metabolic signature (see Results §3.9, Discussion §4.1).

Table S4. NMS leave-one-endpoint-out weight-ranking concordance

left_out	spearman_rho	kendall_tau
F5_ALZHDEMENT	0.966667	0.888889
F5_VASCDEM	0.983333	0.944444
G6_ALS	0.883333	0.722222
G6_ALZHEIMER	0.966667	0.888889
G6_EPLEPSY	0.983333	0.944444
G6_MS	0.916667	0.777778
G6_PARKINSON	1	1
PDSTRICT_EXMORE	0.983333	0.944444

Table S5. Metabolite-specific NMS weight ranges across leave-one-endpoint-out runs

metabolite	NMS weight	leave-one-endpoint-out minimum	leave-one-endpoint-out maximum	leave-one-endpoint-out range
Acetoacetate	0.133976	0.131255	0.139073	0.00781852
Acetone	0.139028	0.12092	0.144318	0.0233979
Citrate_by_Glucose	0.0753331	0.0638745	0.0852239	0.0213494
Creatinine	0.147858	0.143719	0.158848	0.015129
Gln_by_His	0.0969761	0.0908606	0.104184	0.0133237
Glucose	0.117733	0.106889	0.126484	0.0195944
Ile_by_Leu	0.114951	0.0840757	0.123893	0.0398178
Lactate	0.0719237	0.0639058	0.0834811	0.0195753
Lactate_by_Pyruvate	0.102221	0.0950475	0.115181	0.0201333

Supplementary Figure S1. Interpretive category classification, empirical rank clustering, and biochemical-category schematic for the 9-metabolite shared systemic signature.

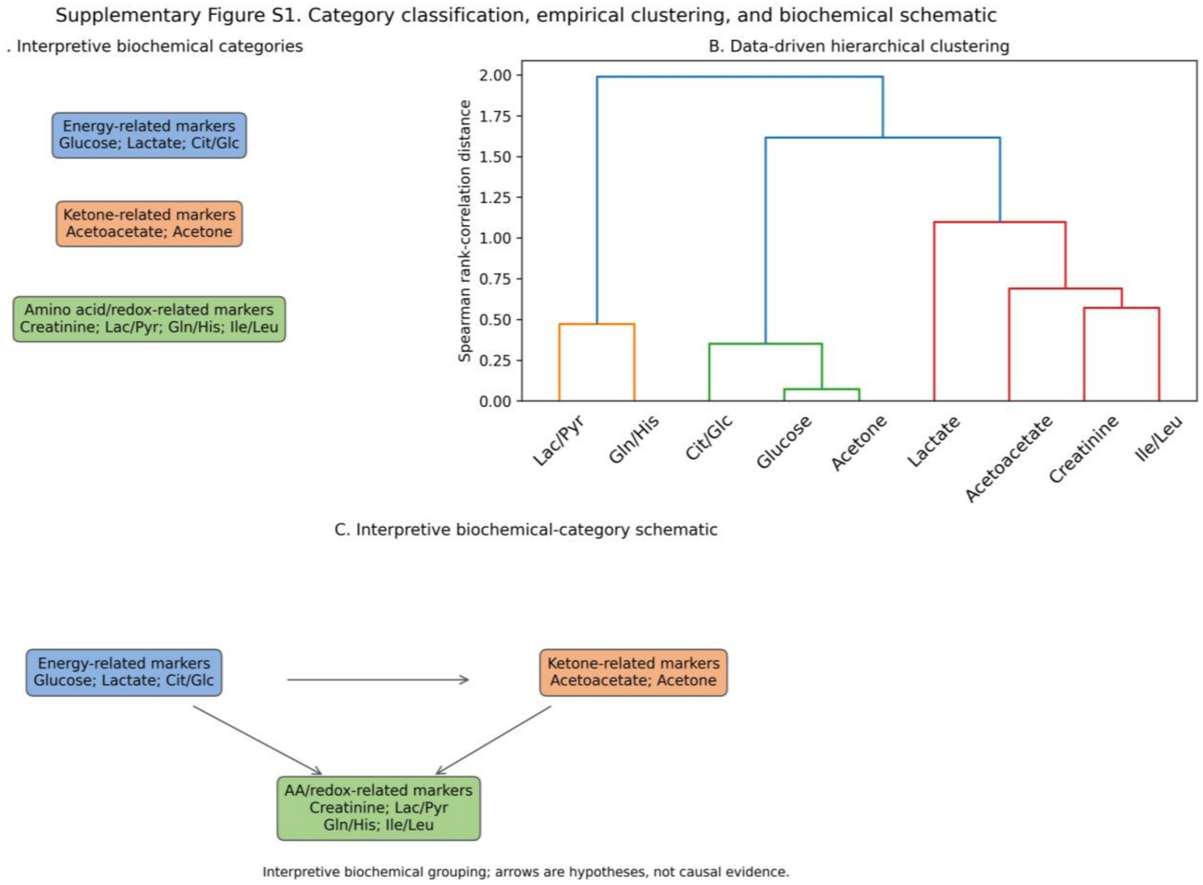

Figure S1. Interpretive category classification, empirical rank clustering, and biochemical-category schematic for the 9-metabolite shared systemic signature: (A) interpretive biochemical classification (energy-, ketone-, and amino acid/redox-related); (B) hierarchical clustering dendrogram (Spearman rank-correlation distance, Ward linkage) across all 8 neurological endpoints; (C) biochemical-category schematic (arrows denote hypotheses, not causal evidence).
